# Evolutionary and structural insights into VP1 epitopes of representative SAT-type FMDV strains: implications for candidate vaccine selection

**DOI:** 10.1186/s13567-025-01643-4

**Published:** 2025-12-15

**Authors:** Shuaiyu Jiang, Shunli Yang, Xiaoqing Zhang, Yupeng Fang, Ziwei Guo, Zhiyong Ma, Xiaoxue Wei, Kuanjia Guo, Ramy E. El-Ansary, Chimedtseren Bayasgalan, Ashenafi Kiros Wubshet, Jie Zhang

**Affiliations:** 1 Hebei Key Laboratory of Preventive Veterinary Medicine, Hebei Normal University of Science and Technology, Qinhuangdao, 066000 Hebei China; 2 Zoology and Entomology Department, Faculty of Science, Al-Azhar University, Cairo, Egypt; 3 School of Veterinary Medicine, Mongolian University of Life Sciences, Ulaanbaatar, 17024 Mongolia; 4 Shandong Binzhou Institute of Animal Husbandry and Veterinary Sciences, Binzhou, China; 5 Department of Veterinary Basics and Diagnostic Sciences, College of Veterinary Science, Mekelle University, Mekelle, Tigray Ethiopia

**Keywords:** Foot-and-mouth disease, SAT serotypes, epidemiology, VP1 protein, vaccine, empty capsid subunit vaccine

## Abstract

Foot-and-mouth disease (FMD) is a highly contagious viral disease primarily affecting cloven-hoofed animals, with outbreaks causing substantial economic losses. This review summarizes the global epidemiological landscape of Southern African Territories (SAT) serotypes of FMD virus (FMDV) from 2010 to May 2025, with a particular emphasis on the topotypic diversity and transboundary spread of SAT2 strains. By integrating phylogenetic analysis and epitope characterization of the VP1 capsid protein, we provide preliminary insights into the evolutionary trajectories of SAT-type FMDVs and their implications for vaccine design. In light of the challenges posed by frequent antigenic drift and poor vaccine matching, we strictly assess the current status of commercial multivalent inactivated vaccines and recent advances in empty capsid subunit vaccine technologies. Comparative evaluation of these strategies underlines the need for enhanced molecular surveillance and rational vaccine optimization to mitigate the ongoing spread of SAT-type FMDV.

**Supplementary Information:**

The online version contains supplementary material available at 10.1186/s13567-025-01643-4.

## Introduction

Foot-and-mouth disease virus (FMDV) comprises seven immunologically distinct serotypes: A, O, C, Asia1, and three Southern African Territories serotypes—SAT1, SAT2, and SAT3—endemic to sub-Saharan Africa. The SAT serotypes, first identified in southern Africa, have long circulated across the African continent and are regarded as a major reservoir of FMDV diversity worldwide. Owing to substantial antigenic variation among the numerous topotypes within each SAT serotype, cross-protection between strains is generally inadequate, posing persistent challenges for vaccine-based control strategies.

Since 2010, SAT-type FMD outbreaks have continued to emerge across Africa. Notably, during 2022–2023, SAT2 viruses belonging to topotype XIV, which originated in East Africa, exhibited transboundary spread into the Middle East and West Asia, including countries such as Iraq, Jordan, and Turkey. This exemplifies the growing risk of transcontinental dissemination, which poses a significant threat to global animal health and disease control [1].

VP1, one of the major capsid proteins of FMDV, harbors key neutralizing epitopes and serves as the principal determinant of antigenicity. Structural analyses have revealed that VP1 contributes to the formation of a conserved surface-exposed cavity on the viral capsid, which may function as a receptor-binding domain and offers a novel target for rational vaccine design [2]. Genetic diversity within VP1 also affects vaccine cross-protection. For instance, a study analyzing VP1 sequences of SAT2 isolates circulating in Ethiopia between 1990 and 2015 demonstrated high genetic variability, particularly within and around the G-H loop, characterized by frequent amino acid substitutions associated with temporal and geographic divergence. These mutations are often localized to critical receptor-interacting regions of the protein [3].

Conventional multivalent inactivated vaccines have shown limited efficacy in countering antigenic variability among SAT-type viruses, especially in the context of emergent topotypes. In response, recent research has focused on the development of novel subunit and virus-like particle (VLP) vaccines incorporating VP1 as a core immunogen. For example, SAT2-type VP1, VP0, and VP3 proteins have been expressed in *Escherichia coli* and evaluated in murine models, where VP1 induced robust humoral and cellular immune responses, reinforcing its potential as a prime target for subunit vaccine development [4]. Furthermore, high-yield production of SAT2 VP1 protein (2.15 g/L) via bacterial fermentation has been achieved, and the refolded protein retained specific antigenicity toward SAT2-specific antibodies. Serological assays confirmed its diagnostic specificity, highlighting its dual potential for both serotype-specific diagnostic assays and safer, more precise vaccine formulations [5].

This review focuses on the global epidemiological trends of SAT-type FMDV from 2010 to 2025, with particular emphasis on the molecular evolution of VP1 proteins from representative topotypes, their antigenic and structural characteristics, and current advances in vaccine development. The insights provided herein are intended to offer both theoretical guidance and practical references for future control strategies and vaccine design targeting SAT-type FMDV.

## Global epidemiological overview of SAT-type FMDV

**Figure 1:**
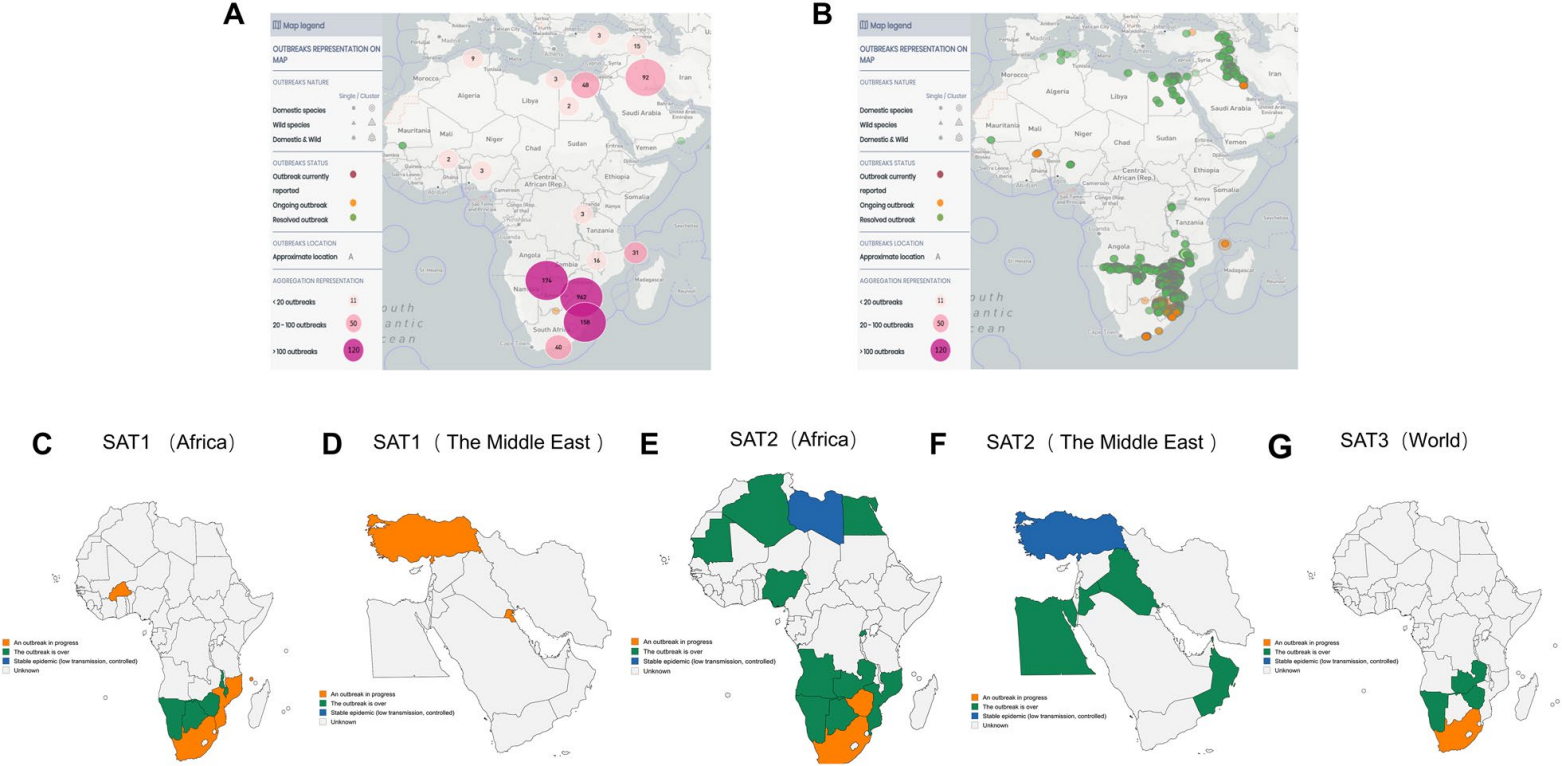
**Global distribution of SAT-type FMD outbreaks (2005–May 2025).** Compiled based on officially reported outbreaks from 2005 to May 2025. **A** Global distribution of SAT-type FMD outbreaks, where the size and color intensity of the circles represent the frequency and scale of reported cases. **B** Spatial point distribution of SAT-type FMD outbreaks, with each dot indicating a confirmed case report; dot density reflects regional reporting frequency and geographic clustering. **C**, **D** Geographic distribution of SAT1-type FMDV, primarily concentrated in southern Africa, with sporadic cases reported in Central Africa and select Middle Eastern countries. **E**, **F** Distribution of SAT2-type FMDV, with outbreaks spanning multiple African regions and extending into parts of the Middle East. **G** SAT3-type FMDV distribution, which remains localized to southern Africa, demonstrating clear regional endemicity. Data source: World Organisation for Animal Health [7]; figure-specific details are specified in the figure.

This study compiled and visualized the global distribution of SAT-type foot-and-mouth disease from 2005 to May 2025, with emphasis on the geographic patterns associated with the three SAT serotypes. As illustrated in Figure 1, SAT1, SAT2, and SAT3 have been predominantly reported in Africa, with the highest number of outbreaks concentrated in southern Africa, marking it as the most affected region (Figures 1A, B).

SAT1 has remained largely restricted to southern African countries such as South Africa, Botswana, and Zimbabwe, although sporadic outbreaks have also been reported in Central Africa, including Burkina Faso (Figure 1C). Notably, in April 2025, the World Organisation for Animal Health (WOAH, formerly OIE)) reported the emergence of SAT1 in Turkey and Kuwait (Figure 1D), drawing international attention to its potential for regional spread.

SAT2 exhibited the widest geographic distribution among the three SAT serotypes. In addition to its presence in southern Africa, it has extended into Central, West, and North African countries and has further spread into the Middle East, including Egypt and Iran. These findings indicate its strong potential for transboundary transmission (Figures 1E, F), underscoring the necessity for proactive surveillance and containment strategies.

In contrast, SAT3 displayed a considerably narrower geographic range, being confined to a few southern African countries such as South Africa, Namibia, and Zimbabwe. Its distribution appeared to be more localized and region-specific (Figure 1G).

In summary, the global distribution of SAT-type FMDV exhibits marked geographic clustering and distinct differences among the three serotypes. Africa remains the primary reservoir of SAT-type viruses. The epidemiological data presented in Figure 1 are based on official outbreak reports released by WOAH between 2005 and 2025 [6].

### Geographical constraints and emerging signals of SAT1 topotype shifts

**Figure 2:**
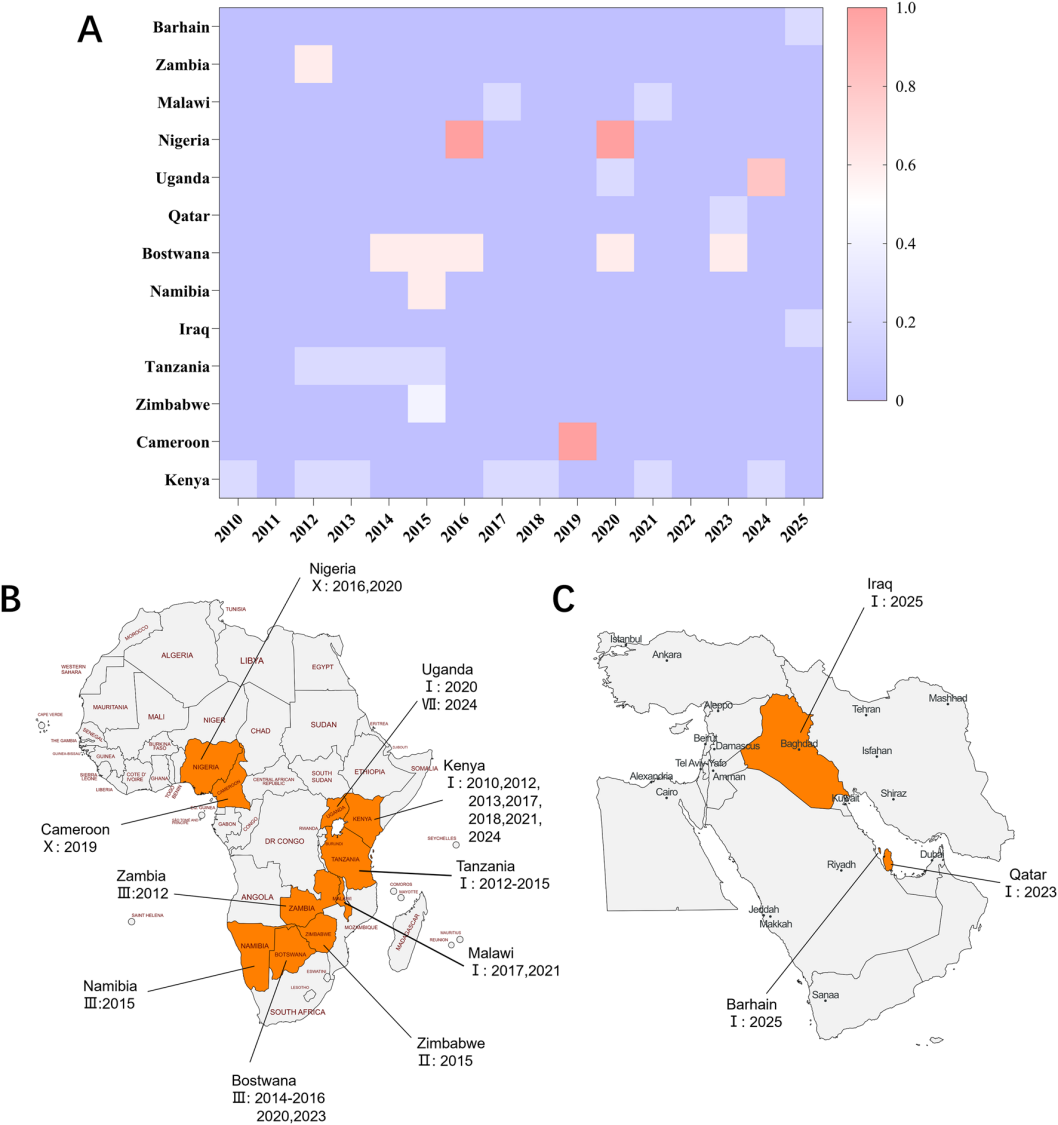
**Spatiotemporal distribution of SAT1 FMDV, 2010–2025.** **A** Heatmap summarizing country-by-year notifications from January 2010 to May 2025. The color scale is a categorical encoding of SAT1 topotypes: 0.2 = I, 0.4 = II, 0.6 = III, 0.8 = VII, 1.0 = X. Colored cells denote years with at least one notification of the indicated topotype; blank cells indicate no notification retrieved for that year. **B** African distribution with country labels showing the reported topotype (Roman numeral) and year(s) of outbreaks. **C** Middle East distribution with analogous annotations. The heatmap reflects reported occurrence, not outbreak magnitude or persistence. Source data for this figure are provided in Additional file 1; figure-specific details are given in the figure.

Historically, the SAT1 serotype of FMDV has been geographically confined to southern and eastern Africa. However, recent epidemiological reports indicate subtle yet significant changes in its spatial distribution. As shown in Figure 2, from 2010 to May 2025, topotype I of SAT1 has been the most widely reported lineage over the past 15 years. Kenya has experienced multiple SAT1 outbreaks between 2010 and 2024, suggesting ongoing viral circulation in the region [8].

Of particular concern, the SAT1 virus was detected for the first time in Iraq and Bahrain in 2025, raising alarms about its transboundary spread. This emergence may be attributed to increased livestock trade or inadequate cross-border disease control measures [9]. In contrast, topotype III has remained localized primarily in southern Africa, with Botswana as the main endemic country. Between 2014 and 2023, several outbreaks were reported in this region, further supporting the notion of persistent viral circulation [10].

Studies have suggested that such circulation may be ecologically linked to wildlife reservoirs. For instance, transmission of SAT1 FMDV from African buffalo (*Syncerus caffer*) to cattle within Zimbabwean wildlife conservancies has been documented, highlighting the role of sylvatic hosts in viral maintenance [11–14].

Meanwhile, topotype VII has only been sporadically detected in Uganda, indicating limited transmissibility and geographic confinement [15]. Although SAT1 exhibits lower antigenic and geographic diversity compared with SAT2, its recent emergence in the Middle East underscores the need for vigilant livestock trade surveillance and the development of region-specific vaccines.

The 2007 equine influenza outbreak in Australia serves as a cautionary example of how insufficient international trade monitoring can lead to the unexpected introduction of animal pathogens. Therefore, the detection of SAT1 in regions previously considered low-risk necessitates proactive surveillance and early vaccine preparedness to mitigate potential outbreaks and associated economic losses [16].

### SAT2: high topotypic diversity and transcontinental spread

Among the SAT serotypes of FMDV, SAT2 exhibits the greatest genetic and geographical diversity, with at least 14 confirmed topotypes [17–19]. This serotype has maintained persistent transmission across sub-Saharan Africa and demonstrated remarkable interregional and transcontinental spread between 2010 and May 2025.

**Figure 3:**
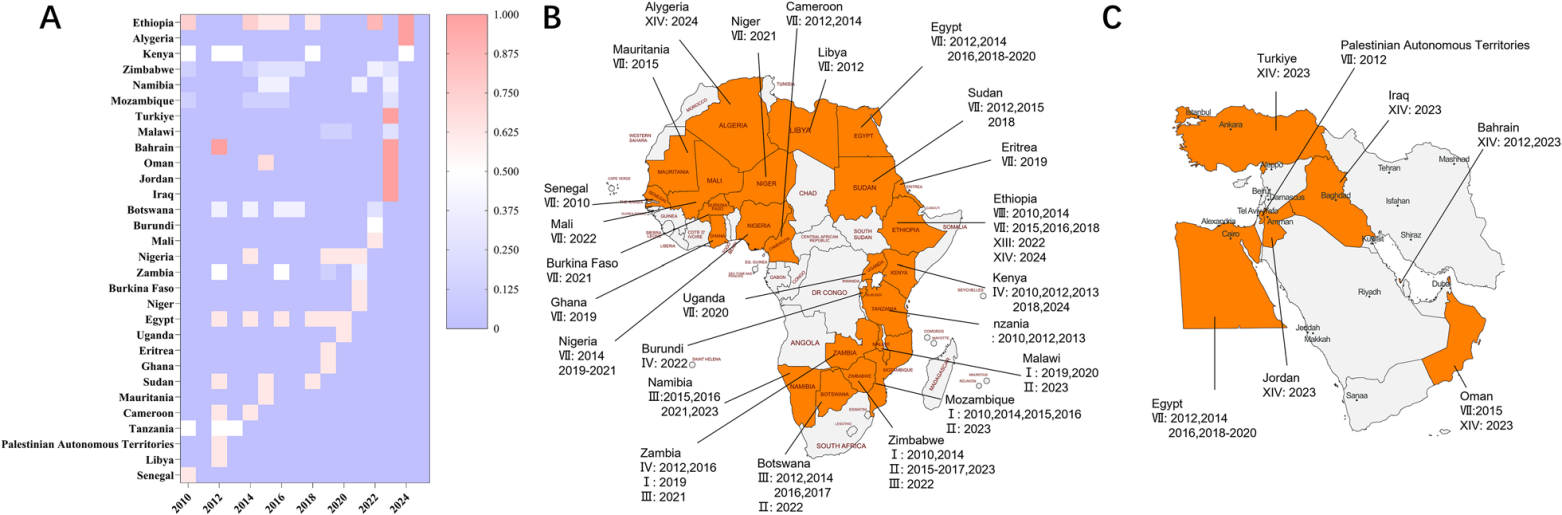
**Spatiotemporal distribution of SAT2 FMDV and topotypes, 2010–2025. A** Heatmap summarizing country-by-year reports from January 2010 to May 2025. The color bar uses a categorical encoding: 0.125 = I, 0.25 = II, 0.375 = III, 0.5 = IV, 0.625 = VII, 0.75 = VIII, 0.875 = XIII, 1.0 = XIV. Colored cells denote years with at least one notification of the indicated topotype; blank cells indicate no report retrieved. Special cases: Uganda (2020) reported both IV and VII, but only VII is displayed to avoid duplication; Ethiopia (2022) reported VII, XIII, and XIV; XIII is shown. **B** African distribution map: countries shaded in orange had ≥ 1 SAT2 notification during 2010–2025; labels indicate reported topotype(s) (Roman numerals) and year(s). **C** Middle East distribution map with analogous annotations. The heatmap indicates occurrence (presence/absence), not outbreak magnitude or persistence; color intensity differentiates category codes rather than severity. Source data for this figure are provided in Additional file 1; figure-specific specific details are given in the figure.

As shown in Figure 3, SAT2 outbreaks have been reported in numerous African countries, including Ethiopia, Kenya, Zimbabwe, and Namibia. Particularly concerning are recent incursions into Jordan, Iraq, Turkey, and Algeria. In 2023, topotype XIV spread from Africa into Jordan and Iraq, marking its first incursion into the Middle East. It subsequently advanced northward into Turkey—a gateway to the European continent—and eastward into Bahrain, highlighting a clear pattern of geographic expansion across the Eurasian landmass [20]. In 2024, the same topotype was detected in Algeria, indicating further dissemination into North Africa [21]. These cross-border transmissions underscore SAT2’s exceptional capacity for long-distance spread, posing significant challenges to regional FMD control efforts.

Over the past 15 years, Ethiopia has reported the co-circulation of multiple SAT2 topotypes, including VII, VIII, XIII, and XIV, suggesting local topotypic coexistence and potential recombination events [22]. Kenya has only sporadically reported topotype IV, with no recent outbreaks recorded. In Zimbabwe, several topotypes (I, II, and III) have been detected in recent years, indicative of sustained endemic circulation. Namibia consistently detected topotype III between 2015 and 2023, although it has remained localized, exhibiting cryptic circulation without evident regional spread [23].

The concurrent circulation of multiple SAT2 topotypes and their evolving distribution patterns substantially complicate vaccine matching efforts. Owing to significant heterogeneity within the VP1 protein, current SAT2 vaccines often fail to confer sufficient cross-protection. This reality highlights the urgent need to strengthen continuous antigenic surveillance systems and develop geographically tailored vaccine strategies to ensure effective immunization coverage and timely outbreak response.

### SAT3: sporadic distribution and low outbreak frequency

**Figure 4:**
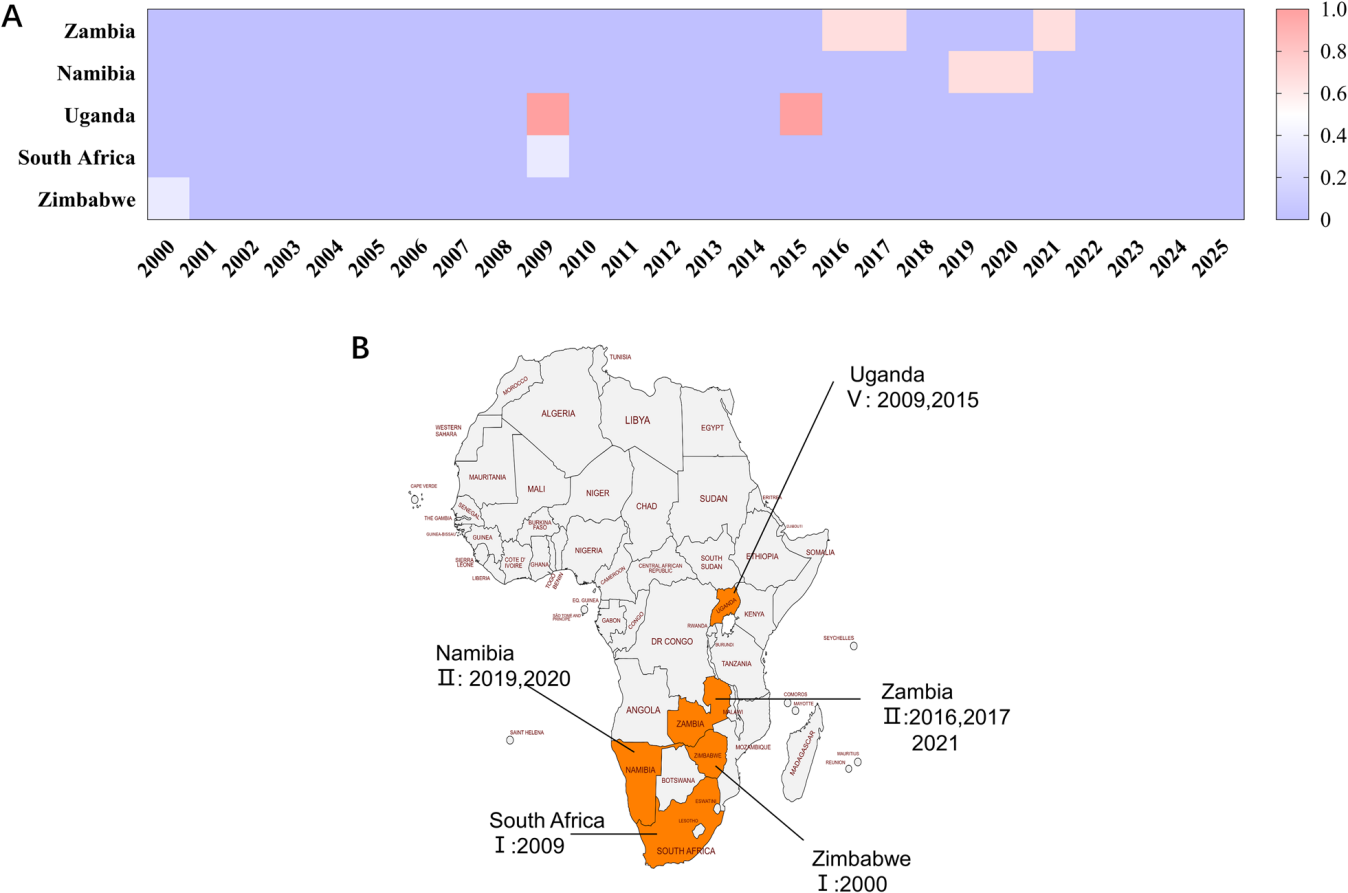
**Spatiotemporal distribution of SAT3 FMDV and topotypes, 2000–2025. A** Heatmap summarizing country-by-year reports from January 2000 to May 2025. The color bar uses a categorical encoding: 0.33 = I, 0.7 = II, 1.0 = V. Colored cells denote years with at least one notification of the indicated topotype; blank cells indicate no report retrieved. **B** African distribution map: countries shaded in orange had ≥ 1 SAT3 report during 2000–2025; labels indicate reported topotype(s) (Roman numerals) and year(s). The heatmap indicates occurrence (presence/absence), not outbreak magnitude or persistence; color intensity differentiates category codes rather than severity. Source data for this figure are provided in Additional file 1; figure-specific details are given in the figure.

As shown in Figure 4, compared with other SAT serotypes, SAT3 exhibits the narrowest geographic distribution and remains endemic only in limited regions of Africa [24]. Between 2015 and 2025, SAT3 outbreaks were sporadically reported, primarily among wildlife and adjacent cattle populations in southern Africa. For instance, outbreaks were documented in Namibia (2019–2020) and Zambia (2017–2018) [25].

SAT3 appears to rely almost exclusively on southern African wildlife, particularly African buffalo (*Syncerus caffer*), as a maintenance host. This has resulted in localized viral circulation within and around protected wildlife areas [26, 27]. Studies have shown that African buffalo can harbor SAT viruses for extended periods without displaying clinical signs, serving as asymptomatic reservoirs [28]. This persistent carrier state may explain how SAT3 periodically resurfaces even in the absence of ongoing domestic livestock transmission. For example, multiple SAT3 isolates have been repeatedly recovered from African buffalo in various wildlife reserves in South Africa and Zimbabwe [28].

Transmission of SAT3 from buffalo to nearby cattle populations represents a critical wildlife–livestock interface risk, particularly where fencing and ecological boundaries are poorly maintained [29–31].

In contrast, SAT3 is rarely reported in East Africa, and its outbreak frequency is considerably lower than that of SAT1 and SAT2. Furthermore, SAT3 demonstrates lower persistence within both primary and intermediate hosts, potentially owing to its shorter duration of infection or maintenance in comparison with the other SAT serotypes [32].

As of 2025, no confirmed transmission of SAT3 outside Africa has been recorded, suggesting strong geographic restriction [33]. The SAT3 virion is also believed to be more susceptible to environmental degradation, with limited survival and transmissibility under natural conditions. As such, its direct impact on the global FMD landscape remains relatively minor [34, 35].Nonetheless, the host specificity and ecological stability of SAT3 imply that, as long as African buffalo populations remain unmonitored and uncontained, complete eradication of SAT3 will remain elusive [36].

In summary, SAT-type FMDV remains predominantly endemic in Africa, with the three serotypes exhibiting distinct differences in spatial distribution, transmission activity, and topotypic evolution. SAT1 is relatively geographically restricted, primarily confined to southern Africa; however, topotype I has recently expanded into the Middle East, indicating a potential shift in its distribution range. SAT2, by contrast, is the most actively spreading serotype, with the broadest geographic coverage and the highest topotypic diversity. Notably, topotype XIV has extended into North Africa and the Middle East in recent years, highlighting its significant transcontinental transmission potential. SAT3 remains highly localized, with sporadic outbreaks confined to wildlife populations in a few southern African countries, and no recorded cases outside the continent to date.

Among the three, SAT2 poses the greatest challenge for control, owing to its high transmission intensity, antigenic heterogeneity, and poor vaccine matching. Moving forward, regionalized control strategies should prioritize continuous surveillance of SAT2 and its dominant topotypes, antigenic evolution monitoring, vaccine strain updating, and emergency stockpiling. Such efforts are essential to address the expanding epidemiological footprint of SAT2 and mitigate the risks associated with its potential intercontinental spread.

## Evolutionary and antigenic characterization of VP1 in representative topotypes of SAT-type FMDV

In recent years, the SAT1, SAT2, and SAT3 serotypes of FMDV have continued to circulate endemically across various regions of Africa, exhibiting pronounced genetic divergence and ongoing antigenic drift. To elucidate their molecular evolutionary relationships and inform vaccine development strategies, this section presents a phylogenetic and antigenic site analysis of the VP1 coding region from key representative strains.

Based on official country-level reports submitted to the World Reference Laboratory for Foot-and-Mouth Disease, the three most frequently reported topotypes within each SAT serotype were selected as representative strains. Their VP1 gene sequences were subsequently analyzed to evaluate evolutionary diversity, antigenic features, and implications for vaccine matching (Figure 4).

Compared with the Euro-Asiatic serotypes (A, O, C, and Asia1), SAT-type FMDVs exhibit substantial sequence variation in VP1, particularly in known antigenic determinants. These unique sequence signatures likely account for the near-complete lack of cross-protection observed both among SAT serotypes and between SAT and non-SAT serotypes [37].

### Phylogenetic analysis of VP1 sequences from representative SAT-type FMDV topotypes

**Figure 5:**
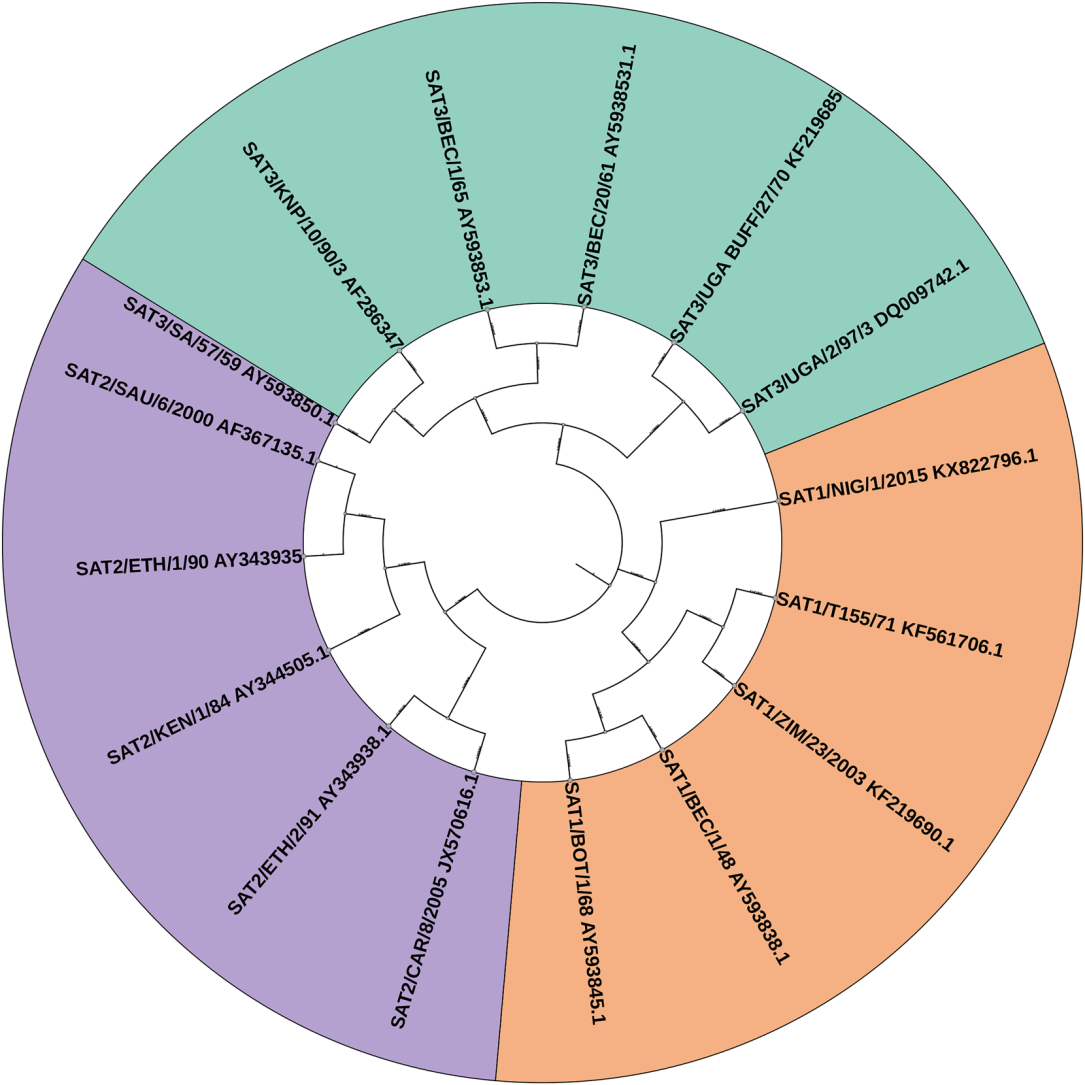
**Phylogenetic tree of representative SAT-type FMDV topotypes based on VP1 gene sequences (neighbor-joining method).** This circular phylogenetic tree illustrates the relationships among representative strains of SAT1, SAT2, and SAT3 serotypes of foot-and-mouth disease virus (FMDV), constructed using full-length VP1 gene sequences. Distinct background colors are used to highlight different topotypes for visualization: SAT1 (orange), SAT2 (purple), and SAT3 (green). One SAT3 strain was mistakenly grouped within the SAT2 cluster owing to topological placement, and this has been indicated in the figure to avoid misinterpretation. Strain labels indicate the topotype, country of origin, and year of isolation, with GenBank accession numbers provided. The tree was generated using MEGA software with the neighbor-joining method under the Kimura two-parameter model. Source data for this figure are provided in Additional file 2; figure-specific details are given in figure.

To systematically elucidate the genetic relationships among representative SAT-type FMDV strains, a phylogenetic tree was constructed on the basis of the full-length nucleotide sequences of the VP1 gene (Figure 5). The results revealed that SAT1, SAT2, and SAT3 serotypes formed distinct, well-supported clades, highlighting their substantial genetic divergence and supporting the notion that SAT serotypes have evolved along separate antigenic and evolutionary trajectories.

Within the SAT1 clade, clear topotypic subdivisions were observed. Topotype I clustered SAT1/T155/71 (KF561706.1) and SAT1/ZIM/23/2003 (KF219690.1), forming an early southern African lineage. In contrast, topotype III included SAT1/BEC/1/48 (AY593838.1) and SAT1/BOT/1/68 (AY593845.1), constituting a distinct southeastern clade. SAT1/NIG/1/2015 (KX822796.1), representing topotype X, emerged as a separate basal branch on the tree, suggesting it may represent a divergent lineage originating from West Africa.

The SAT2 clade displayed greater complexity, reflecting the broader topotypic diversity of this serotype. Topotype IV included SAT2/KEN/1/84 (AY344505.1) and SAT2/ETH/1/90 (AY343935.1), representing the classical East African cluster. Topotype VII, composed of SAT2/SAU/6/2000 (AF367135.1) and SAT2/CAR/8/2005 (JX570616.1), formed a cross-regional cluster encompassing the Middle East and Central Africa [38]. SAT2/ETH/2/91 (AY343938.1), representing topotype XIV, occupied a genetically distant position from other topotypes, suggesting the presence of a novel lineage with potential local adaptation or distinct transmission history.

The SAT3 clade also separated into multiple topotypes. Topotype I was represented by SAT3/SA/57/59 (AY593850.1) and SAT3/KNP/10/90/3 (AF286347.1), both originating from southern Africa. Topotype II included SAT3/BEC/1/65 (AY593853.1) and SAT3/BEC/20/61 (AY593531.1), reflecting intraregional genetic variation within Botswana. Topotype V, consisting of SAT3/UGA BUFF/27/70 (KF219685.1) and SAT3/UGA/2/97/3 (DQ009742.1), originated from Uganda and likely represents an independent evolutionary lineage maintained within East African buffalo populations.

In summary, phylogenetic reconstruction of VP1 sequences from representative SAT-type topotypes not only revealed clear genetic separation among the three SAT serotypes but also clarified the topotypic classification of key outbreak strains. The distinct topotypic clusters reflect complex regional distribution patterns and localized evolutionary pressures across Africa. These findings reinforce the need for regionally tailored vaccine strategies, with topotype-specific antigen selection to enhance cross-protective efficacy and improve the precision of FMDV control programs [39].

### Structural exposure characteristics of core antigenic epitopes in the VP1 protein

To characterize the spatial distribution of key antigenic sites, we selected VP1 amino acid sequences from 15 representative FMDV strains belonging to nine topotypes across SAT1, SAT2, and SAT3 serotypes. Three-dimensional structures of VP1 proteins were reconstructed using the AlphaFold2 prediction model [40]. Subsequently, B-cell linear epitopes were predicted using BepiPred-2.0 from the IEDB suite, and conformational epitopes were identified via ElliPro [41, 42].

Comparison of the two prediction approaches revealed three core epitope regions that were consistently identified across all topotypes and spatially clustered on the protein surface: residues 5–28 (highlighted in yellow) located at the N-terminal tail, highly surface-exposed, and likely involved in initial host receptor interaction; residues 94–101 (orange), mapped to the EF loop region, a known target of neutralizing antibodies; residues 137–144 (pink), situated near the base of the G-H loop, structurally conserved and potentially a broadly cross-protective epitope [43].

**Figure 6:**
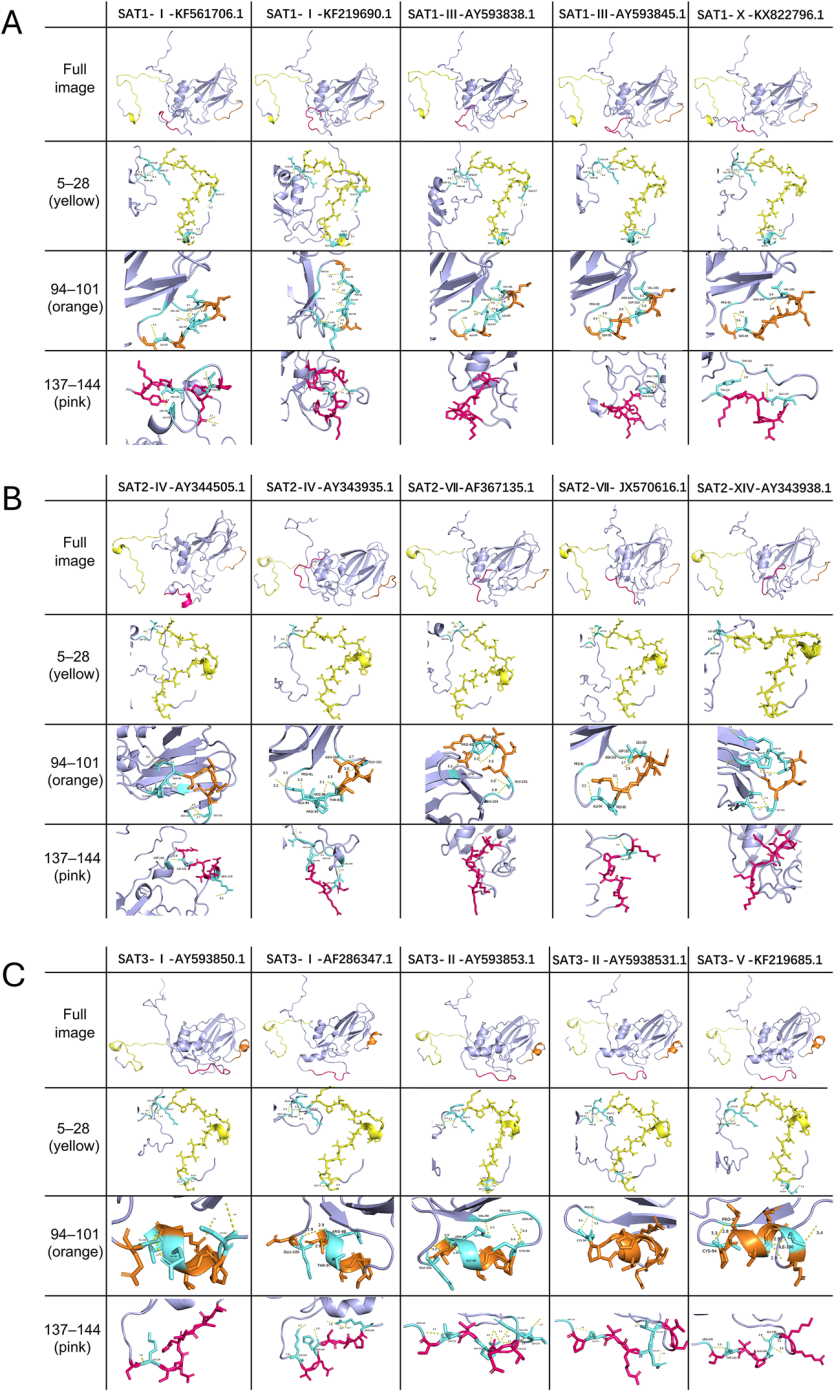
**Predicted 3D structures and B-cell epitope mapping of VP1 proteins from 15 representative SAT-type FMDV strains.** Structures were predicted using AlphaFold2 for 15 FMDV strains covering nine representative topotypes: SAT1 (topotypes I, III, and X), SAT2 (topotypes IV, VII, and XIV), and SAT3 (topotypes I, II, and V). **A** SAT1 strains. **B** SAT2. strains. **C** SAT3 strains. Linear and conformational B-cell epitopes were predicted using BepiPred-2.0 and ElliPro tools from the IEDB resource. Three core epitope regions were identified as recurrent and spatially conserved across all topotypes: residues 5–28 (yellow), located in the N-terminal tail; residues 94–101 (orange), mapped to the E-F loop region; residues 137–144 (pink), positioned at the base of the G-H loop. Each model illustrates the overall VP1 conformation and the spatial distribution of predicted epitopes. Enlarged views highlight local hydrogen bonding interactions and surface accessibility. All structures were modeled and rendered using PyMOL. The results suggest that the three epitopes are surface-exposed and structurally stable across SAT serotypes, supporting their potential as broadly cross-reactive antigenic targets for vaccine development. Source data for this figure are provided in Additional files 3 and 4; figure-specific details are provided in the figure.

Using PyMOL visualization, all 15 VP1 structures were spatially annotated and compared (Figure 6A: SAT1; Figure 6B: SAT2; Figure 6C: SAT3). These three epitopes were consistently found to be exposed on the surface as stable β-turns or loop conformations, indicating good immunological accessibility and structural conservation (Figure 6; or see Additional file 3 for a clearer presentation). Although minor spatial variations in the VP1 backbone were observed among different topotypes, these core epitope regions remained conformationally preserved, supporting their potential as universal antigenic targets for cross-serotype vaccine development.

### Epitope sequence conservation and topotype-specific variation

**Figure 7:**
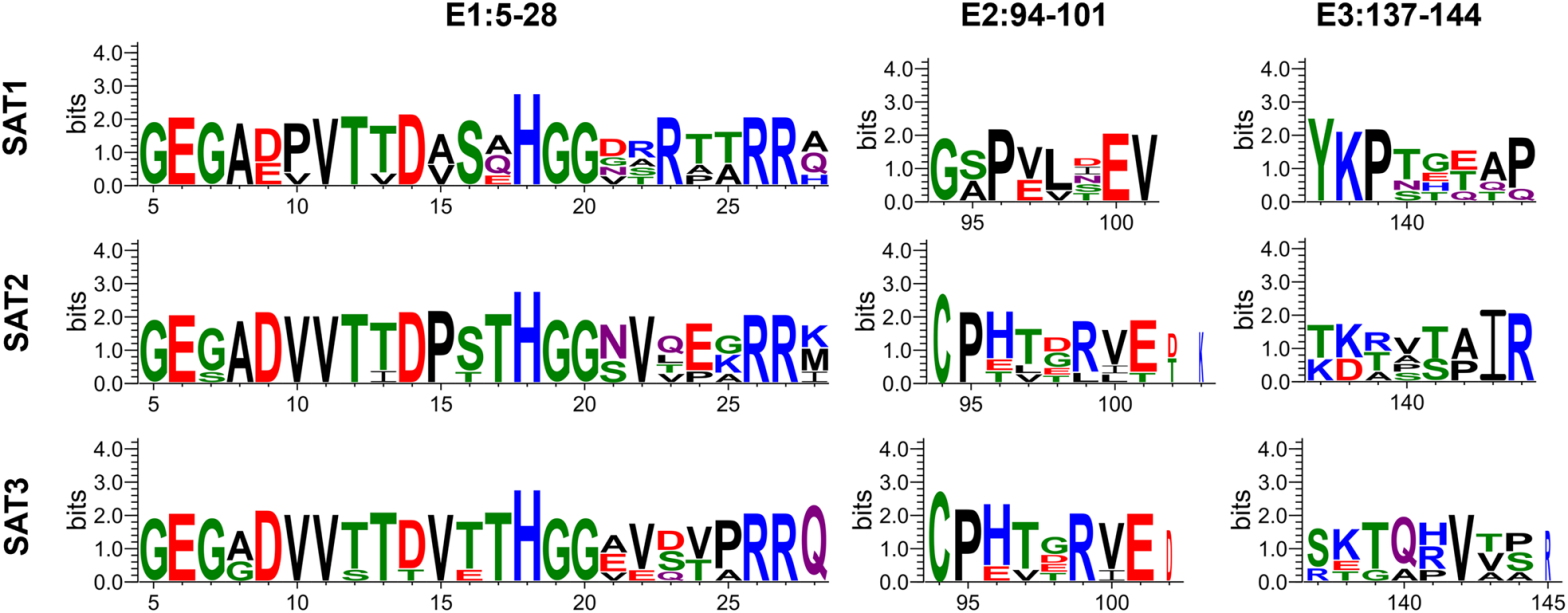
**WebLogo analysis of three core B-cell epitopes across 15 representative FMDV strains from nine SAT topotypes.** The height of each amino acid character indicates the degree of sequence conservation at that position. The three epitope regions correspond to the N-terminal flexible loop (residues 5–28), the E-F loop turn (residues 94–101), and the edge of the G-H loop (residues 137–144). All regions contain several highly conserved residues, while also revealing distinct patterns of sequence variation across different serotypes and topotypes. These results provide a molecular basis for selecting broadly protective epitope candidates for cross-reactive vaccine development. Source data for this figure are provided in Additional file 5; figure-specific details are provided in the figure.

To further assess the conservation and topotype-specific variation of VP1 epitopes among the SAT1, SAT2, and SAT3 serotypes, we focused on three B-cell core epitope regions—residues 5–28, 94–101, and 137–144—identified by both BepiPred-2.0 (linear) and ElliPro (conformational) prediction methods. Multiple sequence alignment of VP1 proteins from 15 representative strains across nine topotypes was performed using WebLogo, allowing for visualization of residue-level conservation and identification of functional sites (as shown in Figure 7).

The first epitope region (residues 5–28), located in the N-terminal flexible tail of VP1, forms a solvent-exposed loop. WebLogo analysis revealed a set of highly conserved residues, including Gly, His, Arg, and Thr, that were consistently preserved across topotypes. This high degree of conservation suggests a potential role in structural stabilization and early-phase immune recognition.

The second epitope (residues 94–101) corresponds to the E-F loop, a well-established site for neutralizing antibody binding. This region displayed moderate overall conservation, with two key residues—Pro at position 95 and Glu at position 99—highly conserved among most topotypes, indicating likely functional importance. In contrast, residues at positions 96, 98, and 100 showed substantial variability, suggesting that they may contribute to antigenic drift. This pattern is indicative of “functional conservation,” in which chemical properties and spatial orientation are maintained despite sequence variation.

The third epitope (residues 137–144) lies at the N-terminal edge of the G-H loop, a region frequently associated with classic FMDV antigenic sites [44]. This epitope exhibited the highest degree of variability among the three, especially in SAT3 strains. The segment is situated near the antigenic ridge and has been identified as a hotspot for antigenic drift [45, 46]. Lysine at position 138 was highly conserved across all topotypes and may serve as a structural anchor, whereas positions 139–141 were markedly variable and likely involved in immune escape mechanisms. Structural modeling via PyMOL showed that this region adopts an outward-facing loop conformation in most cases, which supports its accessibility for antibody recognition. However, its variability also suggests a potential challenge for achieving broad-spectrum protection.

In summary, while all three epitope regions (5–28, 94–101, and 137–144) demonstrated varying degrees of topotype-specific variation, each contains conserved core residues with immunological relevance. Their surface exposure and sequence conservation patterns support their potential use as candidate targets for universal epitope-based vaccines against SAT-type FMDV.

## Advances in vaccine development for SAT-type foot-and-mouth disease

Effective vaccination remains a cornerstone strategy for controlling SAT-type FMD. Given that SAT1, SAT2, and SAT3 serotypes are predominantly endemic to the African continent—with occasional incursions into the Middle East—vaccine research and deployment efforts have largely focused on high-risk regions in these areas. This section provides an overview of the current landscape of SAT-type FMDV vaccines, including both conventional commercial formulations and emerging vaccine technologies. A comparative analysis is presented to highlight the strengths and limitations of each approach in the context of regional control needs.

### Conventional vaccines: application of multivalent inactivated formulations

Owing to the high antigenic variability and geographically distinct topotypic structure of SAT-type FMDV, conventional monovalent or bivalent vaccines often fail to provide adequate cross-protection. Since 2000, widespread circulation of SAT1, SAT2, and SAT3 serotypes across Africa and parts of the Middle East has driven the development and adoption of multivalent inactivated vaccines as the primary regional control strategy [47–49]. These vaccines are typically produced by propagating virus strains in BHK-21 cells, followed by formalin inactivation and formulation with aluminum hydroxide or oil-based adjuvants [50].

Studies have shown that the trivalent SAT vaccine applied in cattle in the Kruger National Park region of South Africa can induce a significant neutralizing antibody response and provide excellent clinical protection in challenge experiments. However, antibody levels decline markedly 4–6 months after vaccination, indicating that booster immunization is required to maintain effective protection [51]. The Botswana Vaccine Institute has developed multiple polyvalent vaccines (SAT1–3) that have been widely used in several African countries. Experimental data demonstrate that the SAT1 vaccine provides good protection with acceptable coverage against circulating strains, whereas SAT2 shows severe antigenic drift, with some strains no longer matched, highlighting the need to update vaccine strains or adopt polyvalent strategies. The SAT3 vaccine exhibits relatively low antigenic matching, with some strains falling outside the protective spectrum, suggesting weaker protective efficacy and the need to improve coverage against newly emerging strains [52]. In addition, the quadrivalent vaccine (A, O, SAT2, and Asia1) developed by MEVAC has been introduced to the market, showing good immune efficacy and protective performance in cattle populations in the Middle East and North Africa [53].

### Emerging vaccine technologies: VLPs and antigenically optimized subunits

With advances in structural biology and recombinant protein engineering, virus-like particle (VLP)-based vaccines have emerged as a promising next-generation platform for FMDV immunization. VLPs are typically generated by co-expressing the FMDV VP1-2A polyprotein and the 3C protease in insect or mammalian cells, resulting in the self-assembly of empty capsids that mimic the native virion structure while lacking viral RNA. These VLPs retain authentic antigenic surfaces and offer superior immunogenicity, safety, and thermostability compared with traditional inactivated vaccines [54–57].

VLP-based vaccines are advantageous owing to their high safety profile, structural stability, and adaptability to differentiating infected from vaccinated animals (DIVA) strategies [58, 59]. For instance, Li et al. developed a SAT2-type VLP vaccine in cattle that achieved 4.33 PD_50_ per dose, exceeding the minimum potency standard recommended by the OIE [60]. Gullberg et al. utilized a single-cycle Semliki Forest virus replicon system to drive co-expression of VP1-2A and 3C in host cells, successfully producing VLPs that elicited strong neutralizing antibody responses in bovine immunization trials [61].

Recent studies have also explored antigenic optimization of VP1, such as modifying immunodominant epitopes or deleting the RGD motif to generate DIVA-compatible vaccines. Patents such as US9457075B2 covering such approaches have been approved in multiple countries [62]. These engineered vaccines exhibit enhanced structural stability, improved thermal tolerance, and greater potential for cross-protection. Although challenges remain in scaling VLP assembly efficiency and yield, the theoretical advantages of this platform are increasingly being realized at both laboratory and pilot production scales.

### Conventional inactivated vaccines versus novel virus-like particle subunit vaccines: a comparative evaluation

To assess the technological trajectory and current limitations in SAT-type FMDV vaccine development, we conducted a systematic comparison between conventional inactivated vaccines and emerging VLP-based subunit vaccines across several critical technical dimensions [63–65] (Table 1).

**Table 1:** **Vaccine comparison: inactivated versus VLP**

Parameter	Inactivated vaccine	VLP subunit vaccine	Evaluation focus
Production method	Requires cultivation of live virus in P3 laboratory facilities	Recombinant expression without viral RNA	Biosafety
Structural stability	Partial capsid disruption; thermolabile	Preserves native-like conformation; thermostability can be engineered	Antigenic integrity
Immunological mechanism	Primarily humoral immunity	Induces both humoral and cellular immune responses	Breadth of immune protection
DIVA compatibility	Most inactivated vaccines are NSP-purified, allowing DIVA differentiation	Genetically engineered for DIVA differentiation	Outbreak traceability
Duration of protection	4–6 months; booster required	≥ 6 months (under optimized protocols)	Antibody persistence
Cost and scalability	Low cost; mature production platform	Higher cost; industrial scalability under development	Technical and economic feasibility

*NSP* nonstructural protein

Inactivated vaccines remain the front-line tool for outbreak control in high-incidence regions owing to their well-established manufacturing protocols and relatively low production costs [66, 67]. However, the increasing frequency of antigenic drift and the growing demand for DIVA-compatible immunization strategies have highlighted the advantages of VLP vaccines. These include improved structural stability, controlled immunogenicity, and customizable design features [68–71], offering a robust foundation for next-generation, precision-oriented vaccine upgrades.

## Conclusions

In recent years, the epidemiological situation of SAT-type FMDV in Africa and its neighboring regions has remained highly complex. Among the three SAT serotypes, SAT2 has exhibited frequent cross-border transmission, posing substantial risks to countries previously unaffected by outbreaks. The SAT serotypes display distinct geographic and genetic divergence in topotypic evolution, VP1 sequence variation, and antigenic architecture, making it difficult for existing vaccines to provide broad-spectrum protection.

In this study, we performed an integrative phylogenetic and epitope-based analysis of VP1 sequences from representative topotypes circulating between 2010 and 2025. Through multiplatform B-cell epitope prediction, we identified a set of conserved immunogenic regions with potential cross-protective value. These findings lay a molecular foundation for the development of next-generation vaccines against SAT-type FMDV.

VLP vaccines based on recombinant empty capsids exhibit strong immunogenicity and safety by mimicking native virions while lacking infectious genetic material. This technology offers a feasible and promising path for future SAT-type vaccine development. To explicitly address manufacturability and purification, several validated and scalable production–purification platforms for FMDV or closely related VLPs can be employed. In bacterial systems (*Escherichia coli*), co-expression of P1-2A with 3C in combination with affinity tags (e.g., SUMO) enables immobilized metal affinity chromatography (IMAC) capture, followed by sucrose cushion/gradient or ion-exchange/size-exclusion chromatography for polishing; this workflow has progressed beyond protection studies in small animals and has been successfully extended to immunization trials in swine and cattle, indicating process transferability from laboratory models to target livestock. Yeast platforms (e.g., *Saccharomyces cerevisiae*) support high-density fermentation of codon-optimized P1-2A-3C constructs and recovery of assembled VLPs through downstream ion-exchange and/or gradient steps, providing a cost-effective alternative to mammalian cells [72]. Baculovirus–insect cell systems (Sf9/High Five) are widely used to express 75S empty capsids; clarified lysates are typically polished by sucrose or CsCl gradients with optional chromatography to reduce host-cell proteins and baculoviral impurities [73]. Plant-based transient expression in *Nicotiana benthamiana* enables rapid, low-cost scale-out; gentle extraction/clarification coupled with two-step cushion/gradient schemes preserves particle integrity and epitope display [74, 75]. Importantly, process and genetic innovations have improved yields and scalability across hosts—most notably the 3C protease L127P variant described by Puckette et al., which reduces cytotoxicity while enhancing P1 processing and VLP formation in mammalian and bacterial cells [76]. Incorporating these unit operations (affinity capture → density-based separation → orthogonal chromatography) together with yield-enhancing strategies into SAT-type programs will strengthen the translational bridge from bench to field without compromising antigenic fidelity.

Going forward, priorities should include antigenic fine-mapping of regional representative strains, cross-serotype protective efficacy evaluations, and DIVA-compatible immunogen design. Together with platform-agnostic controls for particle integrity, host-cell impurity profiles, and batch-to-batch consistency, these strategies will be essential for translating molecular research into field-applicable tools and for establishing a stronger global barrier against the spread of SAT-type foot-and-mouth disease.

## Supplementary Information

**Additional file 1. SAT Country Reports, detailed data in Figures 2-4.**

**Additional file 2. Sequence alignment, detailed data in Figure 5.**

**Additional file 3. SAT structural prediction, detailed data in Figure 6.**

**Additional file 4. SAT B-cell epitope prediction, detailed data in Figure 6.**

**Additional file 5. Core epitopes, detailed data in Figure 7.**

## Data Availability

No datasets were generated or analyzed during the current study.
